# Hereditary thoracic aortic disease associated with cysteine substitution c.937T > G p.(Cys313Gly) in *FBN1*

**DOI:** 10.1007/s12471-019-1296-4

**Published:** 2019-06-12

**Authors:** E. Overwater, K. Van Rossum, M. J. H. Baars, A. Maugeri, A. C. Houweling

**Affiliations:** 1 Department of Clinical Genetics, Amsterdam UMC, University of Amsterdam, Amsterdam, The Netherlands; 2 Department of Clinical Genetics, Amsterdam UMC, Vrije Universiteit Amsterdam, Amsterdam, The Netherlands

**Fig. 1:**
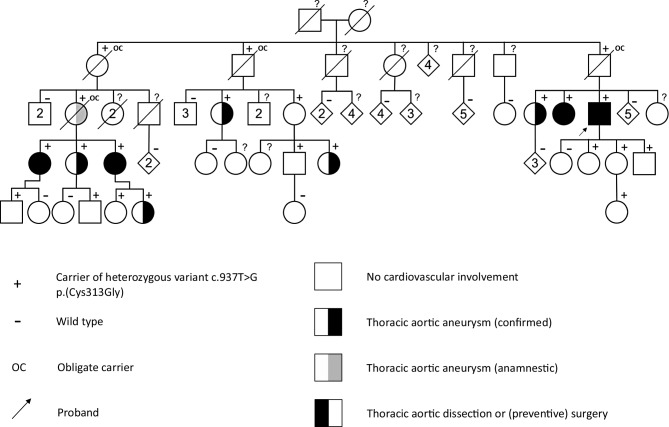
Pedigree depicting thoracic aortic aneurysms and/or dissections. *Squares* represent males, *circles* indicate females

A 56-year-old male was diagnosed with a type A aortic dissection, followed by a type B dissection 3 years later. There were no other signs indicating a familial connective tissue disorder. Pathogenic variant c.937T > G p.(Cys313Gly) in *FBN1* [(NM_000138.4), Online Mendelian Inheritance in Man (OMIM) entry *134797] was identified by DNA testing, consistent with Marfan syndrome (OMIM entry #154700). The variant was identified in 21 out of 53 tested relatives (Fig. 1). A thoracic aortic aneurysm was diagnosed in eight relatives carrying the variant, three of whom met the criteria for preventive surgery. One of the deceased obligate carriers probably had a thoracic aortic aneurysm. Most mutation carriers had a systemic score [1] of zero or one, although the highest score was four. As illustrated by this image, *FBN1* variant c.937T > G p.(Cys313Gly) can cause isolated aortic disease. Timely recognition of individuals with a pathogenic *FBN1* variant is highly important, as it enables the prevention of severe cardiovascular complications [2, 3].
